# 粒子植入治疗联合CT引导下微波消融治疗中晚期肺癌及肺转移瘤的临床分析

**DOI:** 10.3779/j.issn.1009-3419.2020.102.06

**Published:** 2020-06-20

**Authors:** 诚 何, 林海 朱, 旭 林, 舟 安, 望 吕, 坚 胡

**Affiliations:** 310003 杭州，浙江大学医学院附属第一医院胸外科 Department of Thoracic Surgery, the First Affiliated Hospital, Zhejiang University School of Medicine, Hangzhou 310003, China

**Keywords:** 经皮微波消融, 肺肿瘤, 肺转移瘤, 粒子植入治疗, 有效性, 安全性, Percutaneous microwave thermal ablation therapy, Advanced lung cancer, Lung metastases, Seed implantation therapy, Efficacy, Safety

## Abstract

**背景与目的:**

粒子植入治疗及微波消融治疗是无法手术的中晚期肺癌及肺转移瘤患者的一种可行治疗方式，然而两种治疗方式联合应用的治疗效果及安全性报道较少，本研究评估粒子植入治疗联合计算机断层扫描（computed tomography, CT）引导下经皮穿刺微波消融治疗中晚期肺癌及肺转移瘤的临床效果及安全性。

**方法:**

回顾性分析本中心2018年5月-2018年12月接收的21例中晚期肺癌及肺转移瘤患者的临床资料，按照临床治疗方案分为两组，其中行粒子植入治疗的作为对照组，将粒子植入治疗联合CT引导下微波消融治疗的作为实验组，比较两组的患者的近期疗效及操作的安全性。

**结果:**

实验组的有效率为28.57%，对照组的有效率为14.28%，两组患者不良反应发生率差异无显著性（*P* > 0.05）。

**结论:**

粒子植入治疗联合CT引导下经皮穿刺微波消融治疗中晚期肺癌及肺转移瘤的安全性可靠，近期疗效尚可。

## Abstract (en)

**Background and objective:**

Microwave ablation and radioactive seed implantation are therapeutic options for patients with advanced lung cancer and lung metastases lesion who are not surgical candidates. However, reports on therapeutic effectiveness and safety of the two combination treatments methods have been limited to small case series. This research was to assess the clinical outcome and safety of radioactive seed implantation combined with computed tomography (CT)-guided microwave ablation for the treatment of advanced lung cancer and lung metastases lesion.

**Methods:**

The clinical data of 21 advanced lung cancer and lung metastases patients who received treatment at author hospital during the period from May 2018 to December 2018 were retrospectively analyzed. The patients were divided into two groups by whether received extra percutaneous microwave thermal ablation therapy. The short-term effectiveness and safety were analyzed.

**Results:**

The efficacy of control group was 28.75%, and the efficacy of experiment group was 14.28%. The rate of adverse reaction between two groups has no significantly difference (*P* > 0.05).

**Conclusion:**

Seed implantation combined with CT-guided microwave ablation for advanced lung cancer and lung metastases is safety and effective.

肺癌是我国发病率最高的恶性肿瘤，也是癌症相关死亡首要因素^[1]^。虽然手术切除仍是早期非小细胞肺癌（non-small cell lung cancer, NSCLC）的标准治疗手段，但高达30%的I期和II期患者由于副发病变或者身体状况较差被视为非手术治疗的候选者^[2]^。经皮微波热消融疗法（percutaneous microwave coagulation therapy, PMCT）为肿瘤微创医治方法，因其具备的疗效显著、安全性高等特点，近年来已被临床广泛应用于恶性肿瘤治疗中，但此方案常由于肿瘤体积大、形状无规则而造成消融不彻底^[3]^。因此，本中心回顾性分析中晚期肺癌及肺转移瘤患者经粒子植入治疗联合计算机断层扫描（computed tomography, CT）引导下微波消融治疗的近期疗效及安全性。

## 1 资料与方法

### 1.1 一般资料

回顾性分析于2018年5月-2018年12月期间在本中心治疗的21例中晚期肺癌及肺转移瘤患者的临床资料，按临床治疗方案分为行粒子植入治疗的作为对照组和将粒子植入治疗联合CT引导下微波消融治疗的作为实验组，其中对照组7例，实验组14例。纳入标准：经病理或CT平扫确诊的中晚期肺癌或肺转移瘤患者，不能或不愿接受外科手术切除治疗，两组患者及家属自愿签署治疗的知情同意书。联合治疗组中，男性10例，女性4例，年龄51岁-87岁，平均（63.2±9.2）岁，肿瘤直径1.3 cm-7.4 cm，平均（3.8±1.8）cm，病理类型：腺癌6例，鳞癌4例，1例神经内分泌瘤，2例外院病理结果未见，1例结肠癌肺转移。对照组中，男性6例，女性1例，年龄51岁-72岁，平均（65.2±7.6）岁，肿瘤直径1.4 cm-7.4 cm，平均（4.0±2.1）cm，病理类型：腺癌2例，鳞癌3例，2例肝癌肺转移。两组上述基线资料比较无统计学意义（*P* > 0.05）。见表 1。

**1:** 21例患者的临床资料 Clinical data of 21 patients

Clinical date	Number	Percent (%）
Gender		
Male	16	0.76
Female	5	0.24
Age (yr)		
> 60	14	0.67
≤60	7	0.33
Pathology		
Primary lung cancer	18	0.86
Metastatic lung cancer	3	0.14
Tumor size (cm)		
> 3.0	15	0.71
≤3.0	6	0.29

### 1.2 治疗方法

#### 1.2.1 对照组

患者术前均行胸部增强平扫CT对肿瘤进行定位，根据患者疾病的具体情况，制定相关的粒子治疗方案。对术前精神紧张者进行适当安抚或给予适量镇静剂。术中患者取适当体位，监测生命体征，备好胸腔穿刺包，在实时CT扫描下确认粒子穿刺部位，消毒铺巾，1%利多卡因局部麻醉，连接粒子植入枪，植入粒子，手术完成后按压穿刺点10 min-20 min。术后行CT扫描进行粒子剂量验证并观察粒子空间分布，剂量不足者立即或择期补植粒子，同时观察是否出现气胸、出血等并发症，对于发生气胸或出血等症状的患者，根据患者情况行胸腔的闭式引流或机械通气，术后常规给予抗感染及止血治疗3 d。粒子植入治疗全过程给予患者吸氧，并由医护工作者护送回病房。

#### 1.2.2 实验组

根据肿瘤大小、形状、部位及与周围组织的关系，制定粒子植入联合微波消融治疗的方案：病灶位于胸壁上或大血管、大气道周围的部分划为粒子治疗区域，其他病灶的大部分区域为微波消融区域。在实时CT扫描下确认穿刺部位，消毒铺巾，1%利多卡因局部麻醉，根据所计算的进针角度和深度，将微波治疗针穿刺进入肺内病灶，再次扫描CT确定微波消融针位置适当，将功率升至50 W，针尖温度升至90 ℃。根据肿瘤大小及形状连续消融3 min-5 min后停止，消融完毕，让患者屏住呼吸，缓慢退出微波消融针同时消融针道。术后再次行CT扫描，同时观察是否出现气胸、出血等并发症，对于发生气胸或出血等症状的患者，根据患者情况行胸腔的闭式引流或机械通气。在微波消融治疗后1周内进行粒子植入治疗。患者术前行平扫CT进行定位，根据所计算的进针角度和深度置入多根穿刺针，再次扫描CT确定各穿刺针位置适当，置入放射性粒子，退出穿刺针。术后行CT扫描进行粒子剂量验证并观察粒子空间分布，剂量不足者立即或择期补植粒子，同时观察是否出现气胸、出血等并发症，对于发生气胸或出血等症状的患者，根据患者情况行胸腔的闭式引流或机械通气等对症治疗，术后予抗感染及止血治疗3 d。

### 1.3 观察指标

通过CT观察评价疗效，参照世界卫生组织（World Health Organization, WHO）制定的肿瘤病灶分类及疗效评定标准分为4级^[4]^。完全缓解（complete response, CR）：CT显示肿瘤消失或较治疗前或体积缩小 > 70%。部分缓解（partial response, PR）：CT显示瘤体缩小≥30%或病灶中央出现坏死或有液性囊腔形成。病情稳定（stable disease, SD）：CT显示瘤体缩小 < 30%或病灶仍为实性、中央无坏死或囊腔形成。病情进展（progressive disease, PD）：CT显示瘤体体积增加且肿块仍呈实性并侵犯周围邻近组织。疾病控制率=（CR+PR+SD）/总例数×100%。统计两组患者的不良反应发生情况，包括出血、气胸、粒子放射性肺炎、粒子脱落情况。

**1:**
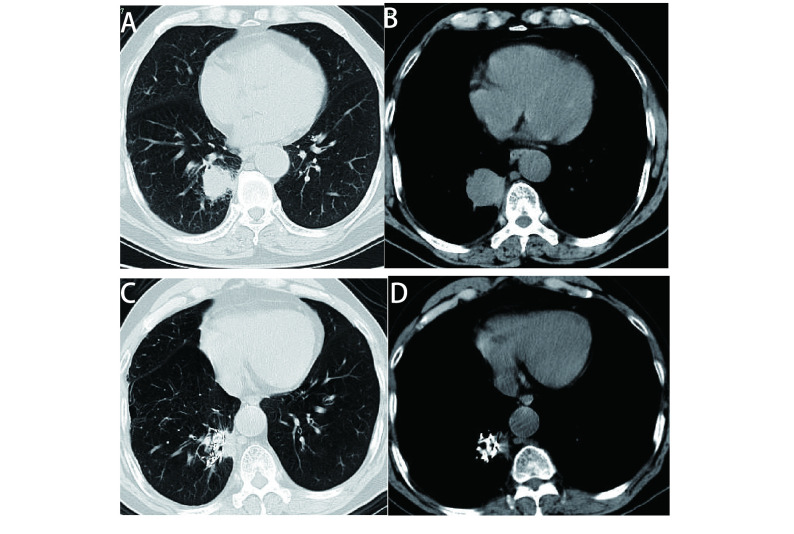
患者男，75岁，右下肺癌。A：CT肺窗示微波消融及粒子植入前的肿瘤病变；B：CT纵隔窗示微波消融及粒子植入前的肿瘤病变；C：微波消融术及粒子植入术后1个月，CT肺窗示肿瘤病灶较前缩小；D：微波消融术及粒子植入术后1个月，CT纵隔窗示肿瘤较前缩小，病灶内见空洞形成。 A 75 year man with right lower lung cancer. A: CT lung window showed tumor lesions before microwave ablation and particle implantation; B: CT mediastinal window showed tumor ablation before microwave ablation and particle implantation; C: One month after microwave ablation and particle implantation, the CT lung window showed a smaller tumor lesion than before; D: One month after microwave ablation and particle implantation, the CT mediastinal window showed that the tumor was smaller than before, and a cavity was formed in the lesion. CT: computed tomography.

### 1.4 统计学处理

采用SPSS 22.0统计学软件对数据进行统计分析。计量资料用均数±标准差（Mean±SD）表示，用t检验；计数资料用率表示，用卡方检验。*P* < 0.05为差异有统计学意义。

## 2 结果

### 2.1 近期疗效

实验组最终纳入14例患者，对照组纳入7例患者，经治疗后，实验组部分缓解4例，对照组部分缓解3例，实验组病情稳定4例，实验组疾病进展6例，对照组疾病进展2例，对照组2例未找到复查结果。粒子联合微波治疗疾病控制率（57.14%）优于粒子单独治疗（42.85%）。

### 2.2 不良反应发生情况比较

治疗结束后，实验组中4例出现气胸，其中3例合并少量胸腔积液。1例气胸合并胸腔积液患者予胸腔闭式引流后好转，其余3例未予特殊处理，自行好转。对照组中1例出现粒子放射性肺炎，1例出现少量胸腔积液。两组患者不良反应发生率差异不明显（*P*=0.701）。

## 3 讨论

中晚期肺癌患者及肺转移瘤患者往往在发现时已失去最佳的手术时机，同时患者的身体状况欠佳无法耐受长期化疗，因而，临床上常以微波、放疗等手段进行治疗。本研究就中晚期肺癌患者及肺转移瘤患者行粒子植入治疗联合CT引导下微波消融治疗的疗效及安全性进行验证。

粒子植入治疗已越来越多的应用于中晚期肺癌患者及肺转移瘤患者，放射性碘-125粒子将低能量γ射线持续释放出来，对肿瘤细胞进行诱导，使其凋亡速度加快，同时对肿瘤细胞DNA合成造成破坏^[5]^。碘-125粒子的穿透能力较低，对于肿瘤周围的正常组织损伤较小^[6]^。然而，尽管单独粒子治疗在中晚期肺癌及肺转移瘤患者上取得了良好的疗效，粒子植入治疗容易引起肿瘤细胞的针道转移，极大地限制了粒子植入治疗的应用，对此，联合射频治疗或者微波治疗可良好地降低针道转移的可能性，在粒子治疗之前，对肿瘤组织进行微波或射频治疗可杀灭肿瘤细胞，在粒子治疗之后，消融针道也降低了针道转移的可能。有报道称，在中晚期肺癌的治疗中，放射性碘-125粒子植入联合射频消融安全可行，能够在极大程度上促进临床疗效的提升^[7, 8]^。与射频消融相比，微波消融需要消融时间短，高温持久，热沉积效应少，消融体积较大，治疗肺部肿瘤优势更大^[9]^。因而采用微波消融联合粒子治疗的优势也越大。

局部热消融术是治疗恶性肿瘤新兴的微创技术，包括经皮射频消融（radio-frequency ablation, RFA）和微波消融（micro wave ablation, WMA）两种，其中WMA应用于早期肺癌治疗，其消融体积更大，复发率低的特点已被众多文献证实^[10, 11]^，然而对于中晚期肺癌和肺转移性肿瘤的疗效报道较少。Wolf等^[12]^报道，CT引导下经皮穿刺微波消融治疗50例患者82个转移性肺癌，74%微波消融部位消融完全，78%的患者肿瘤没有复发，1年局部控制率为67%，微波消融后1年、2年、3年生存率分别为65%、55%和45%。马幸生等^[13]^报道，CT引导下经皮穿刺微波消融治疗术后6个月，所有病灶达到完全缓解（26/45，占57.8%），达到部分缓解（9/45，占20.0%）；总体有效率为77.8%；1年、2年、3年生存率分别为89.5%（34/38）、73.7%（28/38）和63.2%（24/38）。

微波消融术是新兴的肿瘤热疗技术，原理是依靠影像技术引导，将微波天线经皮肤穿刺进入肿瘤内，在微波电磁辐射场作用下，肿瘤中碳水化合物、蛋白质等极性分子和钾、钠、氯离子等带电粒子高速转动，产生摩擦生热效应，肿瘤组织迅速达到70 ℃-160 ℃的高温，造成肿瘤内蛋白质变性，从而使肿瘤细胞发生凝固性坏死^[14, 15]^。完全消融，表现为病灶消失，完全的空洞形成，纤维化进展或疤痕形成，实性结节消失或没有改变和/或肺不张，CT没有增强的征象，被认为是技术效果。不完全消融表现为：不完全空洞形成伴有残留的实性或液性成分；部分纤维化或者是纤维化病灶伴有实性残留物；和/或实性结节大小不变或者是体积增大伴不规则边缘或CT图像有内部增强信号。然而微波消融在消融大气道、大血管旁的病灶时危险性较高，容易引起出血、气胸等并发症，同时，微波消融在治疗球形病灶时效果较好，而对于不规则的病灶往往无法取得满意的疗效，需要多点消融，此时，粒子治疗可对这些缺点进行良好的弥补。粒子植入适用于大气道及大血管旁的病灶，而不会损伤气道和血管，粒子植入联合微波消融可取得较好的疗效。本研究中，粒子植入联合CT引导下微波消融治疗的患者不良反应较轻，经对症治疗后就可好转，发生率和单独粒子治疗组相比无明显差异，进一步证实了粒子植入联合CT引导下微波消融治疗的安全性。

本研究由于受样本量的影响、患者复查原因的影响和随访时间的影响，未能对粒子植入联合CT引导下微波消融治疗的疗效进行良好的对比，待进一步研究。

综上，采用粒子植入联合CT引导下微波消融治疗中晚期肺癌及肺转移瘤患者疗效尚可，可有效达到减瘤的目的，且不良反应较轻，安全性可靠，值得在临床治疗中进一步探索其应用范围及应用价值。
